# Postnatal depression and reproductive success in modern, low-fertility contexts

**DOI:** 10.1093/emph/eow003

**Published:** 2016-03-14

**Authors:** Sarah Myers, Oskar Burger, Sarah E. Johns

**Affiliations:** a School of Anthropology and Conservation, Marlowe Building, University of Kent, Canterbury, Kent CT2 7NR, UK

**Keywords:** postnatal depression, life history, evolutionary demography, fertility, parity progression

## Abstract

**Background and objectives:** Postnatal depression (PND) presents a puzzling phenomenon to evolutionary anthropologists as it is highly prevalent and yet detrimental to child development and maternal health. Adaptive explanations have been proposed, but have not been tested with data that directly link PND to female fertility.

**Methodology:** A survey was designed to gather complete reproductive histories and retrospective measures of PND to measure the effects of PND on fitness. Respondents were born between 1930 and 1967, with the majority based in the UK during their childrearing years. The hypothesis that PND is detrimental to fitness is assessed using Mann–Whitney *U* tests on completed fertility. Binary logistic regression modelling is used to test the hypothesis that PND reduces the likelihood of parity progression.

**Results:** Women experiencing PND at their first or second birth have lower completed fertility, with PND at the first birth leading to lowered fertility. Logistic regression analyses show that this is the result of reductions in the likelihood of parity progression to a third birth when PND is experienced at the first birth or when repeat bouts occur.

**Conclusions and implications:** Our results call into question adaptationist arguments, contribute to the growing understanding of the importance of emotional wellbeing to fertility decision making, and given the economic consequences of markedly below replacement fertility, highlight a potential new source of financial incentive to invest in screening and preventative measures to ensure good maternal mental health.

## Teaser

Postnatal depression reduces completed fertility; women who experience it early in their childbearing careers are less likely to have a third birth. Postnatal depression at the first birth leads to lowered fertility levels, indicating a causal role in population ageing and highlighting a new incentive to invest in prevention.

## BACKGROUND AND OBJECTIVES

Postnatal depression (PND), operationally defined as a depressive episode occurring within 12 months after a birth [1–3], presents a puzzling phenomenon for evolutionary anthropologists because it has detrimental impacts on social, emotional, physical and cognitive development in children [4–9]. These deficits arise from the negative effect that PND has on the quality of mother–infant interaction [10–13]. Because it involves investment in children, emotional stress and condition of the mother, PND should be of great interest for researchers of parental investment or quality–quantity offspring trade-offs. Yet, since pioneering theoretical work by Hagen [14, 15], Thornhill and Furlow [16] and Crouch [17], PND has received very little empirical study leaving open questions as to why this emotional state is so prevalent (a meta-analysis of studies found an average prevalence rate of 13% [18]) and whether it could be adaptive.

Parental investment in an individual offspring is costly, taking up a parent’s energy and time [19]. Parenting prevents investment in other existing offspring, future offspring, or in mating effort, thus there will always be a trade-off between parenting and other activities related to survival and reproduction. Parental investment theory predicts the withdrawal or diversion of parenting when the benefits are outweighed by the costs [19]. Using this framework, Hagen [14, 15] and Thornhill and Furlow [16] have sought to explain PND as an adaptive signal to a mother that she is experiencing a cost to her fitness by investing in a particular offspring and should therefore reduce or eliminate investment [14–16]. Hagen [15] and Crouch [17] further propose that distress displayed by those with PND is also an adaptation to elicit support from kin, thus offsetting costs associated with childrearing. If PND is an aid to maternal investment decision making [14–17], then women in poor circumstances who have PND may be expected to benefit from future reproduction enabled by resources saved or gained from kin, relative to those who do not experience PND.

However, PND also carries a range of costs. It is characterized by active social isolation and refusals of offers of help [20], so is unlikely to be an effective means of enhancing offspring investment through social subsidy. The deficits to child development are indicative of costs to the mother in terms of offspring reproductive potential. If the effects of an episode are confined to just one offspring, it is possible that a mother’s other offspring will be unaffected or benefit in terms of the total investment they receive. Yet, PND is highly recurrent [21], it inhibits a woman’s ability to care for herself and other existing offspring [22, 23], and it predisposes women to future bouts of depression [24]. The occurrence of PND in women in seemingly affluent circumstances is problematic for explanations of PND which frame it as an adaptive aid to maternal investment decisions when circumstances are poor and thus, constrain fitness. Hahn-Holbrook and Haselton [25] have recently put forward an evolutionary based ‘mismatch hypothesis’ for PND aetiology, proposing that it results from a modern parenting environment characterized by low kin support, dietary alterations, early weaning and lack of physical activity. If PND is a disease of modern civilization then its impact on reproductive success would be expected to be detrimental, or at least neutral.

The evidence to evaluate the relationship between PND and fitness is limited and indirect, drawn from studies of depression at other times in the life course. Depression presents major costs to morbidity and mortality, causing prolonged inflammation increasing the risks of various diseases [e.g. see Refs 26 and 27], and heightens suicide risk [28, 29]. The single study that has investigated the impact of general depression on female fertility found compared to a control group depressed women had fewer pregnancies and live births [30]. The physical effects of PND may render women less able to conceive as it alters the hypothalamic–pituitary–ovarian axis [31]. PND becomes chronic in 38% of sufferers [32], and a lifetime history of depression increases risk of earlier menopause [33]. It may also make women less attractive to mates. PND leads to increases in marital problems [34] and depression reduces social attractiveness [35], increases rate of failure for relationships [36–38], and reduces economic prospects [39, 40]. Finally, women may actively avoid childbearing to prevent repeated PND [41].

The evidence on the fitness-related consequences of PND is limited, but strongly suggests that adaptationist explanations are in need of targeted investigation. The lack of data quantifying the effects of PND on fertility is surprising given its likely negative impact [42], especially as PND occurs at relatively high levels in Western countries; estimates range to 63% [43]. We report the results of a survey designed to gather complete reproductive histories and retrospective measures of PND to measure its effect on fitness.

### Hypotheses tested

#### Hypothesis 1ᾦrdquo;PND is detrimental to fitness

Examining the effects of PND on completed fertility indicated that PND was costly, so we tested two further hypotheses to investigate how this effect arises.

#### Hypothesis 2ᾦrdquo;PND reduces the likelihood of progression from the parity at which it is experienced

Multivariate binary logistic regression models are used to assess the effect of PND on parity progression, after controlling for other variables which influence fertility. While we predict that PND will always reduce fertility, we also conduct a moderation analysis to assess adaptive predictions that PND will have a positive effect on the fertility of women in poor circumstances.

#### Hypothesis 3ᾦrdquo;PND will show an additive negative effect on the likelihood of progression from higher parities

We assess (*a*) *the effect of increasing number of bouts* and (*b*) *the effect of PND beyond the parity at which it occurred*. Further, if as the medical literature suggests, PND is costly and causes an additive negative effect, then models accounting for repeat bouts, or effects beyond the parity at which the PND occurred, will be better at predicting parity progression than models in which a bout of PND is only considered as an independent event as implied by adaptive accounts. To test this prediction, we compare the models from *Hypothesis 3* with those from *Hypothesis 2*. For the same reasons the effect sizes of the PND measures utilized in *Hypothesis 3* should be larger, because they are cumulative, than those used in *Hypothesis 2*, and this prediction is also assessed.

## METHODOLOGY

### Data collection

Complete reproductive histories of post-menopausal women were collected by retrospective questionnaire. Respondents reported details about every birth they had experienced and were assessed on a number of demographic and psychological measures. Participants were recruited via advertising in newsletters and social media channels of UK-wide branches of the Women’s Institute [44], alumni networks of two UK universities and social media aimed at older women. The survey was conducted online using SurveyGizmo and, to minimize inaccurate reporting due to the nature of information requested, participants remained anonymous with the exception of their IP address to control for multiple responses from the same address: 306 valid responses were received. Data are available from the Dryad Digital Repository: http://dx.doi.org/10.5061/dryad.cf6nh.

### Measures

#### Postnatal depression

Women self-reported their PND history in three ways: whether they had received an official medical diagnosis, the Bromley Postnatal Depression Scale (BPDS) [45] and a modified Edinburgh Postnatal Depression Scale (EPDS) [46]. PND is notoriously under-diagnosed [47] and retrospective use of the BPDS and EPDS provided valuable additional screening.

The BPDS consists of a statement regarding depressive symptoms and a question regarding whether such symptoms were experienced; if the answer is affirmative their duration is recorded, with anything over a month indicating PND. This was used to determine a categorical measure of PND incidence at a given parity. The BPDS is designed to assess PND symptoms retrospectively [45] and has been used in studies assessing similar durations of recall [48, 49], yet it provides no scope for assessing severity of symptoms. For this reason, we use a modified version of the EPDS.

The 30-point EPDS is the most widely used screen for PND [50]. Questions were presented in the past tense and participants were requested to reflect back on the first year after each birth. To the best of our knowledge this is the first application of this form of the EPDS retrospectively over a long-recall duration, but it has been used retrospectively over 5 years [51]. An alternatively modified EPDS has also been used as part of the Netherlands Study of Depression and Anxiety (NESDA) to assess lifetime prevalence of PND [52]. The EPDS score for each birth was used as a continuous measure of *PND severity*. A categorical measure of PND incidence after each birth was determined using a cut-off score of 12 following Payne *et al.* [51] and the NESDA [52]; this is a higher cut-off than suggested by Cox *et al.* [46] and deemed appropriate due to the accuracy of recall in retrospective reporting of depression increasing with severity [53]. Finally this measure of incidence was used to determine a continuous measure of *PND history*, i.e. the number of PND bouts up to and including a given parity.

In addition to PND, the other measures used within the regression analyses can be seen in Table 1. These include demographic and sociological controls, along with measures that are especially influential in the probability of parity progression, and a measure of general depressive tendency throughout the life course.

**Table 1.** Measures taken retrospectively from 306 post-reproductive women

**Variable**	**Measure/description**	**Reason for measuring/influence on parity progression**
Dependent		
Parity progression	Was there a subsequent birth? Yes/No (categorical)	
Predictors		
PND	Actual diagnosis, BPDS, EPDS (see main text) (categorical/continuous)	Hypothesized to negatively influence parity progression
Age at previous birth	Age at birth in years. Year of offspring’s birth minus year of mother’s birth (continuous)	To control for fertility decline with age [54]
Breastfeeding	Were the offspring breastfed? Yes/No (categorical)	Suppression of ovulation is short-lived [55, 56] and it may enhance experience of motherhood due to improved attachment [57, 58]
Depressive tendency	Depression score from the Depression Anxiety Stress Scales short version [59]. Trait wording is used to assess depressive tendency throughout the adult life course [60]. Possible scores range from 0 to 42 (continuous)	Negatively influences CFR [61]
Emotional experience of birth	Rate the emotional experience of this birth. Positive/Mixed/Negative (categorical)	Birth trauma impacts maternal wellbeing and willingness to undergo future pregnancies [43, 62]
Infant birth weight	Was birth weight normal? Birth weight classified as ‘normal’ or ‘not normal’ (low or high) (categorical)	Low birth weight increases CFR [63, 64] and high birth weight at increased risk of future morbidity [65–67]
Infant health	Did offspring have any serious health issues in their first year? Yes/No (categorical)	Poor health increases CFR [63, 64]
Physical experience of birth	Were complications experienced at this birth? No complications/Minor complications/Major complications (categorical)	Complications likely to reduce the likelihood of parity progression [68, 69]
SES during childbearing years	Social Class Based on Occupation method [70] Participants classified occupation of household member contributing majority of finances. SES either high (professional), medium (managerial and technical), or low (skilled non-manual, skilled manual, partly skilled and unskilled) (categorical)	To control for any effects of SES
Social pressure to be a good mother	Did you experience social pressure to be a ‘good mother’? Yes/No (categorical)	Perception of social stigma associated with stress and depression [71, 72], so likely to increase negative affect and alter fertility desires
Support from family	Rate the level of support in offspring’s first year High/Medium/Low (categorical)	Kin network influences female fertility decision making in contemporary Western populations [73, 74], that peer support may prevent PND [75], and that social isolation is linked to depression [71]
Support from friends	Rate the level of support in offspring’s first year High/Medium/Low (categorical)	As above
Support from mother	Rate the level of support during pregnancy and offspring’s first year. None indicates respondent’s mother was not alive at time of first reproduction. High/Medium/Low/None (categorical)	As above
Support from offspring father	Rate the level of support in offspring’s first year. High/Medium/Low (categorical)	As above
Year of mother’s birth	Year of mother’s birth (continuous)	Controlled for any confounding effects of the respondents being born during a period of fertility decline [76]

### Sample characteristics

Respondents were born between 1930 and 1967, and their average age was 59.1 years (SD 7.5). The majority of respondents (82.3%) were married throughout their childbearing years, of high to medium socioeconomic status (SES) (‘professional’ 68.0%, ‘managerial and technical’ 20.6%), with the women’s husband/partner contributing the majority to household finances (77.1%). The majority did their childrearing in the UK (73.9%), followed by the USA (12.8%). On average, respondents gave birth to 2.28 infants (range 1–6). For the percentage of the sample that continued childbearing at each parity and the distributions of each measure of PND across parities, see the supplementary material.

### Data analysis

#### Hypothesis 1

Completed fertility was used as the main fitness-relevant fertility measure to evaluate the impact of PND. We compared respondents who had experienced PND at least once with those who did not and then respondents who experienced PND in association with a specific parity level (1–3) with those who did not; a Mann–Whitney *U* test on completed fertility was conducted for each group.

#### Hypotheses 2 and 3

Binary logistic models assessed the likelihood of parity progression from parity 1–2 (*P1*), 2–3 (*P2*) and 3–4 (*P3*), with the exception of *Hypothesis 3a* when only P2 was analysed owing to inadequate sample size (see supplementary material for details). Progression to greater parities was not analysed because very few women in the sample had more than four births (*N* = 3).

To test *Hypothesis 2*, we fitted models for each parity that increased in complexity based on the number of variables included in the generalized linear model. The first *PND only model* estimated how PND severity alone affected parity progression. Second, a *base model* controlled for the effects of *year of mothers birth*, *age at birth* and *SES*. Third, a *full model* including all possible variables in Table 1 was run. While we had theoretical reasons (see Table 1) to enter all of our covariates at once into our analysis, the results from the *full model* (see supplementary material for details) found the influence on parity progression of numerous variables to be either entirely neutral or variable by parity. Therefore, we then created a *selected model* in which forward stepwise selection searched for the strongest predictor variables at each parity from the full selection of variables (Table 1), to which we then added, if excluded, PND (to track its effects) and the variables *year of mothers birth*, *age at birth* and *SES* (to control for demographic effects).

The same procedure was utilized to test *Hypotheses 3a* and *b* (for the resulting *selected models*, see supplementary material). In *Hypothesis 3a* the measure of interest was *PND history*, i.e. the number of bouts of PND experienced. The effect of *PND severity* at parity 1 on progression from P2 and P3 and the effect of *PND severity* at parity 2 on progression from P3 were the measures of interest in *Hypothesis 3b*.

#### Effect sizes and model comparison

Akaike’s information criterion with a second order bias correction (AICc) is used to compare models across *Hypotheses 2–3b*. Additionally, continuous variables were centred and standardized and reported in the supplementary material. This not only removes some of the potential for collinearity but it also makes the regression coefficients interpretable as effect sizes because the units have been removed and the variance standardized.

#### Moderation

We tested for moderation at each parity level as part of *Hypothesis 2* by testing for interaction effects between *PND severity* and each of our categorical covariates (Table 1), controlling for *age at birth* and *mother’s year of birth*. We also created a continuous measure of a mother’s circumstance at a given parity, reflecting the number of ‘poorest’ categories a mother was rated in for each of the covariates. A score of 1 was assigned if the mother fell into the following categories: minor or major *birth complications*, not *breastfeeding*, negative *emotional experience of birth*, abnormal *infant birth weight*, *infant health issues*, low *SES*, low *support from family*, *friends*, the offspring’s *father*, and low or no *support from their mother* (*social pressure* was excluded due to the poorest category choice being debateable). The scores were summed and used as a continuous numerical variable with a possible range of 0–10. Using this measure we tested for an interaction between *maternal circumstances* and *PND severity*, again controlling for *age at birth* and *mother’s year of birth*. Variables were centred and standardized before performing the moderation analysis.

All statistical analysis was conducted using R (v.3.2.1).

## RESULTS

### Hypothesis 1

When parity was not taken into account respondents who experienced PND at least once showed a non-significant trend towards lower completed fertility (Table 2). When PND experience at different parity levels was assessed, respondents who experienced PND at their first birth had lower completed fertility compared with those who did not according to all measures of PND, as did those with PND measured by the EPDS at their second birth (Table 2). Those with PND measured by the EPDS at their third birth had lower completed fertility at a level approaching significance.

**Table 2.** Mean number offspring born dependent on PND experience, standard error (SE), 95% confidence intervals (CI) and Mann–Whitney *P* (one-tailed) values for tests on the difference in completed fertility dependent on experience

**PND experience**		**PND measure**					
		**BPDS**		**EPDS**		**Actual diagnosis**	
		Mean offspring no. (SE) (95% CI)	Mann– Whitney *P*	Mean offspring no. (SE) (95% CI)	Mann– Whitney *P*	Mean offspring no. (SE) (95% CI)	Mann– Whitney *P*
PND at least once	No	2.313 (0.058) (2.200–2.427)	0.104	2.280 (0.062) (2.158–2.401)	0.397	2.291 (0.053) (2.186–2.395)	0.297
	Yes	2.178 (0.090) (2.003–2.354)		2.269 (0.081) (2.109–2.428)		2.220 (0.124) (1.970–2.469)	
PND at first birth	No	2.332 (0.055) (2.224–2.440)	**0.002**	2.347 (0.058) (2.232–2.462)	**0.004**	2.302 (0.052) (2.199–2.404)	**0.017**
	Yes	1.936 (0.083) (1.770–2.103)		2.076 (0.086) (1.905–2.247)		1.964 (0.120) (1.717–2.211)	
PND at second birth	No	2.541 (0.051) (2.450–2.642)	0.075	2.567 (0.054) (2.461–2.673)	**0.008**	2.524 (0.048) (2.429–2.619)	0.164
	Yes	2.372 (0.100) (2.170–2.574)		2.328 (0.083) (2.161–2.494)		2.391 (0.151) (2.079–2.704)	
PND at third birth	No	3.300 (0.060) (3.181–3.419)	0.596^a^	3.338 (0.066) (3.205–3.470)	0.053	3.311 (0.061) (3.191–3.431)	0.404^a^
	Yes	3.333 (0.333) (1.900–4.768)		3.077 (0.077) (2.909–3.245)		3.000 (na) (na)	

a Exact test used due to small sample size.

The *P* values are in bold.

### Hypothesis 2

The direction of the effect of increasing *PND severity* at a given parity on progression from that parity was not consistent across parity levels (Table 3). The point estimate for the effect of increasing EPDS score at parity 1 was non-significant for each model but always negative. At parity 2, there was a significant negative effect in models with EPDS on its own and after controlling for demographic factors; the effect remained negative yet lost significance once more factors were controlled for. At parity 3 the negative effect found when EPDS was on its own and after controlling for demographic factors shifted to a positive effect once more factors were controlled for, although all results were non-significant and our sample size is small (*N* = 92 at parity 3). The full regression results for each model, including the effect sizes for each variable, are provided in the supplementary material.

**Table 3.** OR for the effect of PND on parity progression across models testing Hypotheses 2–3b

**Model**	**Variable of interest**		**Progression from parity 1**				**Progression from parity 2**				**Progression from parity 3**			
			OR	AICc	*R* ^2^_CS_	*R* ^2^_N_	OR	AICc	*R* ^2^_CS_	*R* ^2^_N_	OR	AICc	*R* ^2^_CS_	*R* ^2^_N_
*Hypothesis 2*														
1 PND only	PND severity at birth *n*		0.963	256.691	0.007	0.012	0.952^**^	316.376	0.021	0.029	0.967	97.510	0.005	0.008
2 Base	PND severity at birth *n*		0.976	233.031	0.110	0.189	0.937^**^	303.230	0.104	0.143	0.947	97.487	0.102	0.155
3 Full	PND severity at birth *n*		1.000	250.032	0.177	0.305	0.947*	324.978	0.174	0.237	1.066	128.112	0.316	0.479
4 Selected	PND severity at birth *n*		0.984	222.176	0.166	0.287	0.966	299.595	0.156	0.214	1.075	91.467	0.230	0.348
*Hypothesis 3a*														
1 PND only	PND history	Bouts x1					0.774	315.389	0.033	0.045				
		Bouts x2					0.290^**^							
2 Base	PND history	Bouts x1					0.700	303.203	0.112	0.153				
		Bouts x2					0.240^**^							
3 Full	PND history	Bouts x1					0.786	324.314	0.185	0.252				
		Bouts x2					0.256^**^							
4 Selected	PND history	Bouts x1					0.791	297.538	0.148	0.203				
		Bouts x2					0.236^**^							
*Hypothesis 3b*														
1 PND only	PND severity birth 1						0.929^**^	312.404	0.037	0.051	0.999	97.952	0.000	0.000
2 Base	PND severity birth 1						0.922^**^	300.635	0.114	0.156	0.995	98.264	0.094	0.143
3 Full	PND severity birth 1						0.907^**^	321.328	0.195	0.266	0.965	135.991	0.317	0.481
4 Selected	PND severity birth 1						0.915^**^	292.806	0.172	0.235	0.996	91.585	0.251	0.380
1 PND only	PND severity birth 2										0.978	97.648	0.003	0.005
2 Base	PND severity birth 2										0.979	98.070	0.096	0.146
3 Full	PND severity birth 2										1.029	135.991	0.317	0.481
4 Selected	PND severity birth 2										0.961	91.162	0.210	0.318

The PND only model contains only the PND measure listed under variable of interest, the Base model contains the additional variables age at birth, mother’s year of birth and SES, the Full model contains all the additional variables listed in Table 1, and the Selected model contains the variables retained after forward selection on the full set of variables after forcing the retention of PND and the Base model variables (see supplementary material for details). PND severity ORs reflect unstandardized results (for effect sizes see supplementary material). AICc shows the relative information loss across models at each parity, and Cox and Snell’s (*R*^2^_CS_) and Nagelkerke’s (*R*^2^_N_) pseudo *R*^2^’s estimate the variance captured by the models **P < 0.05, *P < 0.1.

### Moderationᾦrdquo;Hypothesis 2

Only two significant interactions were found (*P *< 0.05) in 60 possible interactions assessed and so we resign the full results of the moderation analysis to the supplementary material. The significant interactions were between *PND severity* and having *support from the infant’s father* (*low* vs *high*) and *PND severity* and the respondent’s *emotional experience of birth* (*mixed* vs *positive*) at parity 2. Further, there was no significant interaction between the combined *maternal circumstances* variable and *PND severity*. The interaction between *PND severity* and *father support* was significant (*P *= 0.047); separating women by level of support found that when women received *high support* the effect of increasing *PND severity* on parity progression had an odds ratio (OR) of 0.898 (*P * = 0.000), and when women received *low support* it was 1.063 (*P *= 0.321) (see supplementary material for full details). The interaction between *PND severity* and *emotional experience of birth* was significant (*P *= 0.005); in women with a *positive emotional experience* the effect of increasing *PND severity* had an OR of 0.901 (*P *= 0.001), and when they had *mixed emotions* the OR was 1.070 (*P *= 0.204).

### Hypothesis 3a

Experiencing more bouts of PND (*PND history*) decreased the likelihood of progressing from parity 2 (Table 3); this was significant across all models. The full results for each regression model can be found in the supplementary material.

### Hypothesis 3b

**Figure 1.**
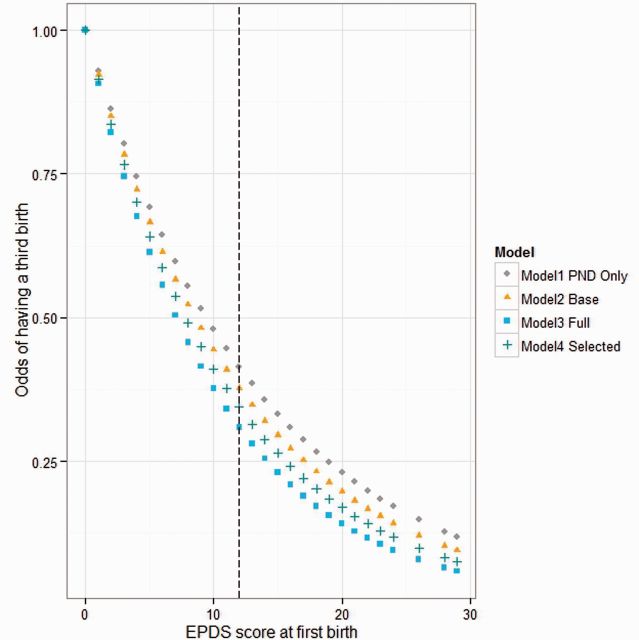
Odds of a third birth at parity 2 dependent on PND severity (EPDS score) at the first birth across all models. The dashed vertical line indicates the cut-off beyond which PND is deemed to have occurred.

Higher *PND severity* at the first birth was associated with decreasing likelihood of progressing from parity 2 (Table 3, Fig. 1); this effect was significant across all models. The effect of higher *PND severity* at either the first or second birth on progression from parity 3 did not reach significance. The full results for each regression model can be found in the supplementary material.

### Model comparison

**Figure 2.**
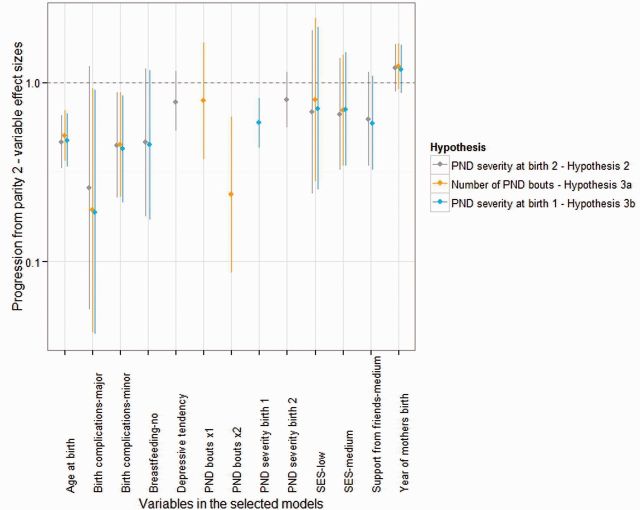
OR plot showing the effect sizes for the impact of variables in the ‘selected models’ on progression from parity 2. Error bars reflect 95% confidence intervals. Continuous variables have been standardized and centred.

The effect of PND was found to be significant in various models at parity 2 across *Hypotheses 2–3b*. Comparing the AICcs of the strongest model (the *selected models*) generated under each hypothesis at parity 2 showed the model containing *PND severity at birth 1* (*Hypothesis 3b*) to lose the least information (Table 3), followed by *PND history* (*Hypothesis 3a*); AICc weights found there to be a probability of 0.863 that the *Hypothesis 3b* model was the strongest (see supplementary material for full calculations). When only *PND severity at first birth* was entered at parity 2 it had an OR of 0.929, falling to 0.915 after controlling for *age at birth*, *year of mother’s birth*, *SES*, *birth complications*, *breastfeeding* and *support from friends* in the *selected model*. The negative effect of *PND severity at birth 1* on progression from parity 2 was of a similar effect size to *age at birth*, and within the range of *minor birth complications* (Fig. 2). Having *a bout of PND at both first and second birth* had the second largest effect size on progression from parity 2, smaller yet within the range of *major birth complications* (Fig. 2). The full list of effect sizes for all variables in each regression model can be found in the supplementary material.

## CONCLUSIONS AND IMPLICATIONS

This study is the first empirical test of the effects of PND on fitness. By showing that PND at the first or second birth is associated with lower completed fertility, and that increasing number of bouts of PND and higher PND severity at the first birth reduce the likelihood of a third birth, our study identifies potential pathways by which PND is detrimental to fitness. These results call into question existing evolutionary explanations of PND based on its having adaptive value and contribute to the growing understanding of the importance of emotional wellbeing to fertility decisions [77].

PND at parities 1 and 2 was found to be costly when analysing completed fertility, being significantly associated with reductions in fertility. Repeat bouts of PND and PND at the first birth are particularly costly, producing the strongest models, and show effect sizes comparable to factors with well-documented influence on fertility such as birth complications [68, 69]. We suggest impacts on parity progression are more strongly seen after two bouts due to the physical or emotional costs of PND being additive. Alternatively, the impact of repeated PND on offspring quality is too great to risk a third bout or the additional costs of a third child. That PND at the first birth has a stronger negative impact on progression from parity 2 than parity 1 is also indicative of its reducing a mother’s capacity to cope with increasing numbers of offspring. Of the women in our sample who had a second birth, roughly 50% of women experiencing PND at their first birth also had it at their second (see supplementary material), mirroring the general population [21]. Depression has a priming effect on the immune system, causing epigenetic changes that lower stress reactivity thresholds, increasing the likelihood of future bouts [78]. PND is as likely, if not more likely, to be experienced at the first birth, raising the probability of repeat bouts if childbearing continues and also increasing the likelihood of depression at other points in the life course.

In terms of evolutionary trade-offs between current versus future offspring, PND appears to be costly. Low fertility strategies in modern post-industrial societies do not result in increased reproductive success in descendants [79], so there are unlikely to be longer term gains from the lower fertility of women with PND. Humans have been found to follow quality–quantity offspring trade-offs in a number of societies [80–83]. PND poses risks to the mother and her offspring, and if taken at face value it would seem unlikely that these women are benefiting in terms of reproductive success from higher quality offspring. However, ceasing to reproduce could provide protective benefits to existing offspring whose level of maternal investment, already impoverished by PND, would be further reduced by the addition of siblings.

These results may reflect PND just being maladaptive in contemporary environments [17], where fertility behaviour in general is not fitness maximizing [84]. Model comparison indicated that the effect of PND is cumulative, suggesting a physical cost is incurred, even in contemporary populations, in line with medical literature [26, 27, 31–33]; it is unclear why the physical costs of depression to health and reproductive function would not be detrimental in past environments. Crouch suggests that in the dense social settings of small-scale societies maternal distress would be quelled by support before it developed into depression [17]. Little research has been conducted on depression in small-scale societies; yet recent findings in the Tsimane, Bolivian forager-horticulturalists, run counter to the notion that depression is simply one of modernity’s by-products [85]. If the effects on fertility are psychological rather than physical in origin, then PND may simply increase the use of contraception and abortion in modern environments. However, cross-cultural data on infanticide and child abandonment are consistent with the optimization of available resources for reproductive effort [86, 87]; if potential future offspring are avoided by postnatally depressed women in contemporary developed settings via increased use modern birth control, then unavoidable offspring born to postnatally depressed women without access to contraception seem likely candidates for experiencing much heightened risk of infant death.

We did not find complete support for all our hypotheses, and do not have adequate data to fully examine the effect of PND at higher parities. We cannot rule out the possibility that it has a positive effect on parity progression likelihood at level 3 and beyond. Our moderation analysis does provide limited support for adaptationist explanations of PND in that its effect was found to be fitness neutral in women experiencing low support from their offspring’s father and a mixed emotional experience of birth at parity 2. However, for the most part our results are not supportive of the adaptive explanations proposed by Hagen [14, 15], Crouch [17], Thornhill and Furlow [16], with the vast majority of our moderation models finding no interaction between PND and circumstance. That PND significantly reduces the chances of progression from parity 2 in women who had high levels of paternal support or positive emotional experiences of birth also raises the question as to why women of such good circumstances become depressed in the first place, and how PND can occur in such women and reduce fitness. Our results do not preclude ‘mismatch hypotheses’ [25] or maintenance based adaptive explanations of PND such as the Pathogen Host Defence hypothesis [88] and the related psychobiological model of depression and social rejection [71]. It has been proposed that PND is a product of particular sociocultural environments [16, 17, 89]. It is possible that, in contemporary developed populations at least, PND is a product of stress responses to low investment under certain circumstances, masking the benefits of a current versus future trade-off. PND may not be an evolved signal to cease investment, but instead be the by-product of responding to some other signal of threatened fitness.

Women diagnosed with PND at their first birth had lower completed fertility than those who were PND free. Factors which contribute to completed fertility are of importance due to the widespread nature of below replacement fertility in the developed world [76]. Below replacement fertility leads to ageing population structures with problematic dependency ratios [90]. Older age structures present major challenges to health and social security systems, potentially inhibit gains in productivity, may negatively impact relations between generations, and reduce social cohesion [91], leading governments to search for ways to raise fertility levels [77]. With PND prevalence around 13% [18], and reaching 63% [43], our results indicate measures to safeguard maternal mental health would be effective as means to increase fertility. Implementation of preventative measures is currently lacking for PND, yet effective strategies are known [92]. In the UK, routine screening is not recommended [93] as it does not prove cost effective [47]. Were PND to be accepted as a factor contributing to below replacement fertility, and thus a causal factor in population ageing and the economic burden this entails [90, 94], the financial costs and benefits of prevention would undoubtedly change.

Unmeasured factors that might be important to our results include abortions, miscarriages, or illness, which may impede fertility. Such factors undoubtedly affected some women, yet for this to be a substantial issue they would have to have disproportionately affected women with PND. Marital/long-term partnership status throughout the reproductive lifespan was not taken into account, however from an evolutionary perspective this can be taken as a proxy for underlying mate quality, for which we had other measures such as depressive tendency. A drawback of our dataset is that we cannot control specifically for level of educational attainment, which is known to influence fertility [95]. However, due to our methods of respondent recruitment, we are confident that the majority of our sample was educated to at least university undergraduate level. A major pathway by which education affects fertility is in the shifting of childbearing to older ages [96], and we did control for age at childbirth in all models with controls. SES is highly positively correlated with educational attainment [97] and this is also controlled for. The use of the EPDS as a retrospective measure of PND may capture women who would not be clinically diagnosed with depression if showing symptoms today; screening measures generally find higher rates of PND than are diagnosed [43], and retrospective assessment it likely to introduce some recall bias. While specific depressive symptoms are more likely to be forgotten than incorrectly reported as having occurred [53], prospective assessment of PND and its effect on progression to subsequent parities may provide stronger causal evidence. Finally our premise, based on medical and psychological literature, was that PND was costly, and thus unlikely to be an adaptive signal to a woman that she is too low on resources to continue investing. Therefore, it is particularly interesting to see what effect PND has in contemporary, developed populations where costs may be borne more easily. However, future research should be aimed at assessing how the results vary across other social and economic contexts.

This study, to our knowledge, represents the first evidence regarding the curtailing impact of PND on female reproductive decisions, and adds to findings emphasizing the importance of parental wellbeing [77]. The results, in combination with the culturally widespread nature [2] and high prevalence of PND, indicate the importance of factoring in women’s emotional experience of early motherhood to demographic models of fertility. Future research is needed to clarify the effect of PND at higher parities, ascertain the cross-cultural range of these findings, and also further assess the influence on fertility of depression at other points in the life course. The effect of PND on fitness-relevant measures other than fertility, such as offspring quality, also needs exploring. Importantly, given the economic consequences of markedly below replacement fertility, our results highlight a potential new source of financial incentive to invest in screening and preventative measures to ensure good maternal mental and emotional health.

## SUPPLEMENTARY DATA

Supplementary data are available at *EMPH* online.

**Conflict of interest:** None declared.

## Supplementary Material

Supplementary Data
